# Pneumococcus Peritonitis

**Published:** 1904-12-17

**Authors:** 


### PNEUMOCOCCUS PERITONITIS.

THE differentiation of cases of peritonitis according to the nature of the infective agent is a matter that has come sharply to the front in recent years, chiefly because it is now realised that proper and efficient treatment will depend largely upon a knowledge of the variety of organism which has caused the inflammation. The recognition for therapeutic purposes of only two forms of infective peritonitis —the tuberculous and the rest—will it is realised, no longer be sufficient. A careful analysis of a number of instances of peritonitis which have been shown by bacteriological examination to be due to a particular organism generally reveals a clinical type which is to some extent peculiar and characteristic. In a paper in the *Annals of Surgery* for December, Dr. F. S. Mathews reports and comments on five cases of pneumococcal peritonitis which have recently been under his observation. The patients were all children between the ages of two and five years, and they all died. In only one case does the peritonitis appear to have been recognised during life, and in this instance laparotomy was performed. In four of the cases empyema was found, in two there was pneumonia, and suppurative pericarditis was also present in one. Discussing pneumococcal peritonitis as a clinical entity, Dr. Mathews points out that it is three times more frequent in children than adults, and while in the former it occurs much more commonly in the female sex, the ratio being 7 : 1, yet in adults the sexes are about equally affected. The pathological changes in the peritoneum are similar to those met with in pneumococcal empyema; that is to say, there is a very fibrinous, greenish-yellow exudate without odour. The consistency of the exudate varies, in one case being practically solid, in another quite fluid, and containing masses of fibrin. There is a decided tendency for the process to become localised in the pelvis, where a large, thick-walled abscess may be formed, and, if this occurs, operation offers a good chance of recovery, but when the inflammation does not become localised a fatal issue is almost certain. In uncomplicated cases, where localisation occurs, three stages may be distinguished. (1) Sudden onset with high fever, vomiting for a day or two, tenderness and distension. There is little muscular rigidity, and the pain and distension are not so marked as in the other types of acute peritonitis. (2) After a few days vomiting ceases, and the temperature often falls to normal. Diarrhœa is usually present. The amelioration of symptoms is pronounced, the appetite returns, and the child looks better. (3) There then appears a tense cystic mass in the hypogastrium, the temperature rises, and symptoms of pelvic abscess appear. The diagnosis is not always easy. In the early stage it may be doubtful whether a peritonitis exists at all, since distension and pain are not necessarily marked, and, even if diarrhœa be absent, as is not always the case, the bowels move readily with cathartics. During the second stage the clinical picture is not unlike that of typhoid, while the late stages are liable to be mistaken for tuberculous peritonitis. The most characteristic features appear to be the absence both of distension and of severe constipation, and the softness of the abdomen in spite of the fact that pain and tenderness are present.

